# A Clinicopathologic Study of Seven Cases of Orbital Solitary Fibrous Tumours

**DOI:** 10.7759/cureus.8259

**Published:** 2020-05-24

**Authors:** Xiao Wei Ting, Shankari Sothiraghagan, Wan Mariny W Md Kasim, Julieana Muhammed

**Affiliations:** 1 Ophthalmology, Universiti Sains Malaysia, Kelantan, MYS; 2 Ophthalmology, University Putra Malaysia, Putrajaya, MYS; 3 Ophthalmology, Hospital Serdang, Kajang, MYS

**Keywords:** solitary fibrous tumour, ocular tumour, immunohistochemistry staining

## Abstract

Objective

To describe the patient demographics, clinical findings, investigations, surgical outcomes, and histopathological findings of seven cases of orbital solitary fibrous tumours.

Method

This was a retrospective review of seven cases of orbital solitary fibrous tumour, which were followed up in Hospital Serdang, a national oculoplastic centre, from years 2008-2017.

Results

This study included seven patients with ages between 21 and 35 years old; two were males and five were females. All seven patients presented with painless chronic unilateral proptosis. Radiological imaging of the orbit showed a localized contrast enhancing intraorbital mass. All patients underwent orbitotomy and excisional biopsy. Intraoperative findings showed a well-encapsulated and vascularized mass. Histological findings of spindle-shaped cells were noted. All cases had positive staining for cluster of differentiation (CD) 34, five were positive for CD 99, four were positive for B-cell lymphoma (BCL-2), and five patients had positive staining for S-100. Three of the patients did not have clear margins during the primary operation and subsequently had a recurrence within two years.

Conclusion

A solitary fibrous tumour is a rare mesenchymal tumour with a pleural origin. The orbit is the most common extrapleural site of the tumour and they are usually benign. Immunohistochemistry is important to differentiate it from other, more aggressive forms of orbital tumours. Regular follow-up is important to monitor for recurrence.

## Introduction

A solitary fibrous tumour (SFT) is a rare mesenchymal tumour, initially described with a pleural origin. The most common site of the tumour is intrathoracic. However, multiple sites of extrathoracic SFT have been reported in recent years, and this includes the liver, nasal cavity, orbit, thyroid, meninges, skin, kidney, and other organs [1]. The first case of an orbital solitary fibrous tumour was described in 1994 by Westra et al. [2]. Since then, only a handful of cases have been reported. There are no published cases of an orbital solitary fibrous tumour in Malaysia thus far. Here, we describe seven cases of orbital solitary fibrous tumours over a period of nine years, along with their surgical outcomes.

## Materials and methods

This was a retrospective review of seven cases of orbital solitary fibrous tumours who underwent surgery and were followed up in Serdang Hospital, a national oculoplastic centre from years 2008-2017. We reviewed the patients’ demographic data, clinical presentation, imaging, surgical outcome, histopathological findings, immunohistochemistry staining and recurrence of the tumour. Institutional review board approval was not required for the present study.

## Results

**Figure 1:**
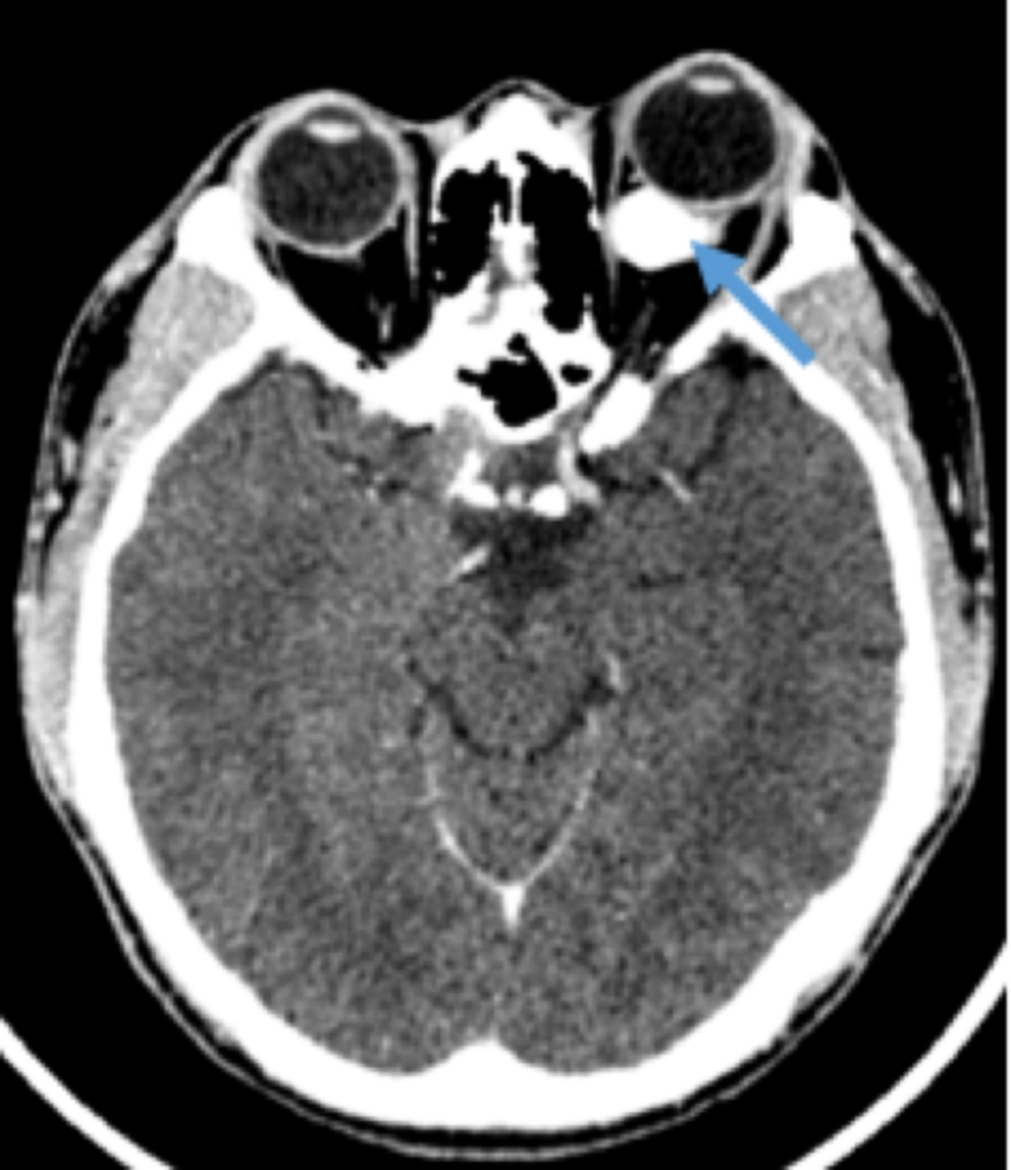
Axial view of a computed tomography image showing a well defined enhancing intraconal mass

Among the seven patients, five were females and two were males. Their age ranged between 21 and 35 years. Clinically, all patients presented with unilateral painless proptosis of more than one-year duration. Table 1 summarises the demographics of the patients and the characteristics of the tumour. Radiological imaging demonstrated a contrast-enhancing lesion that displaced the globe. The lesions were either intraconal or extraconal but localized to the orbit. Although two patients had a mass effect on the extraocular muscles and optic nerve, there was no direct involvement of the optic nerve (Figure 1). There was no systemic involvement in all of the cases.

**Table 1:** Patient demographics and tumour characteristics

CASES	GENDER	AGE	RACE	LATERALITY	VISUAL ACUITY	TUMOUR LOCATION	TUMOUR SIZE
CASE 1	Female	32	Chinese	Right	6/12	Superior Temporal, Intraconal	3 x 5cm
CASE 2	Female	35	Malay	Left	6/9	Superior Nasal, Extraconal	2 x 2cm
CASE 3	Female	25	Malay	Right	6/12	Superior Nasal	3 x 2cm
CASE 4	Female	22	Malay	Left	6/6	Nasal, Extraconal	2 x 2cm
CASE 5	Male	32	Malay	Right	6/9	Temporal, Extraconal	4 x 2cm
CASE 6	Male	21	Malay	Left	6/6	Intraconal	3 x 2.5cm
CASE 7	Female	21	Malay	Right	6/6	Retro-orbital, Intraconal	3 x 3.5cm

**Figure 2:**
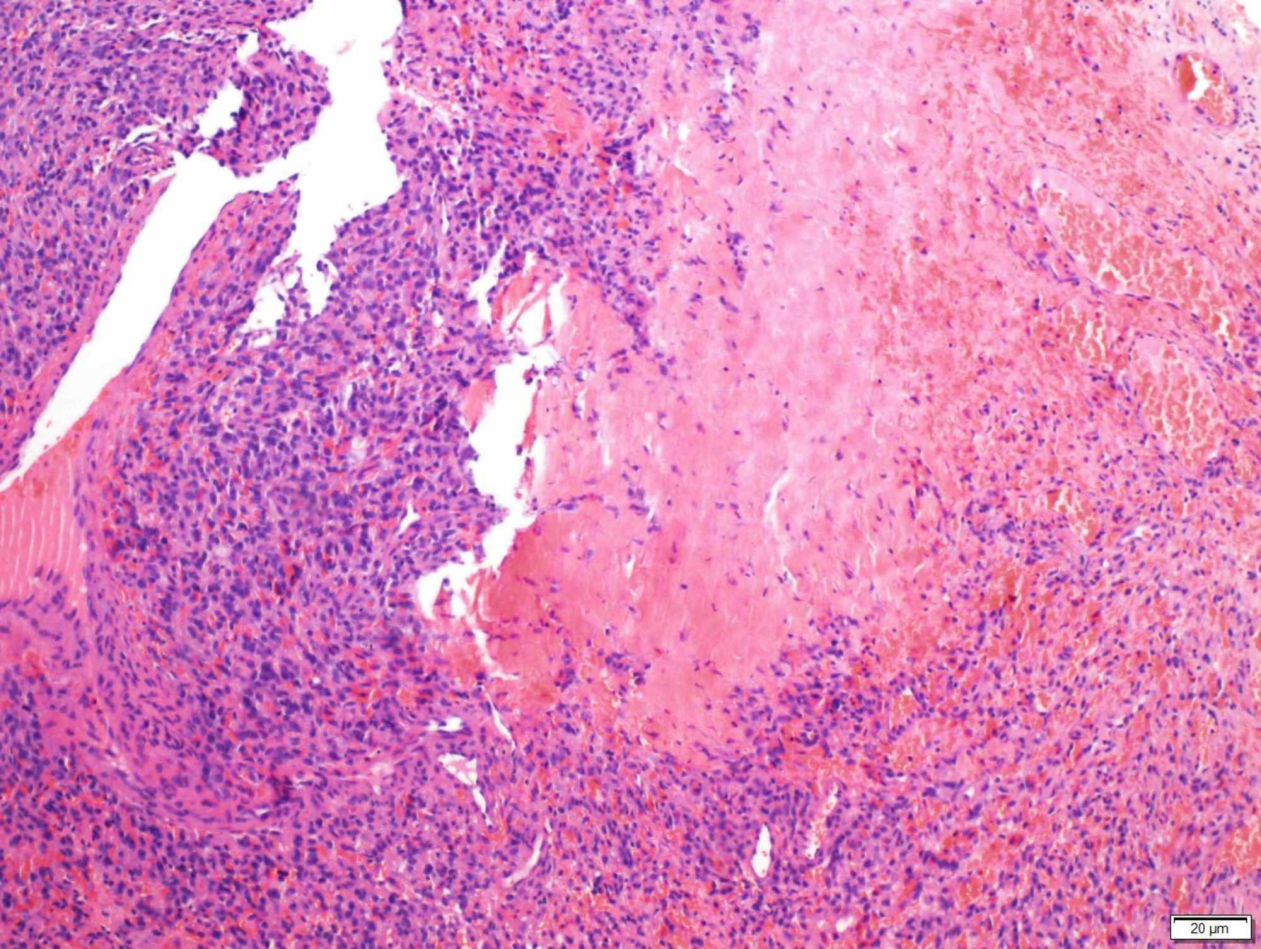
Round and oval spindle cells in fibrous stroma

**Figure 3:**
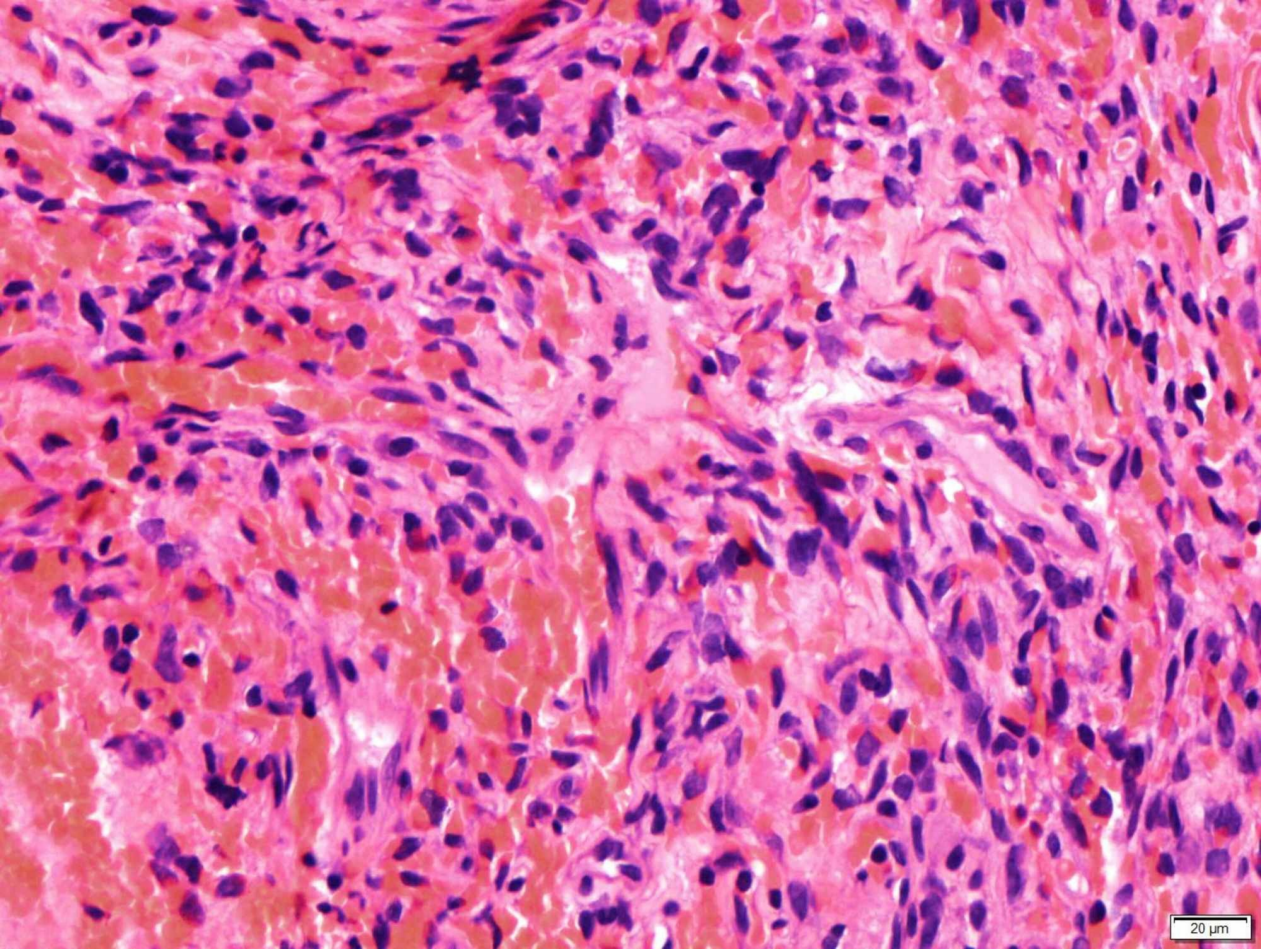
Thin-walled branching blood vessels

**Figure 4:**
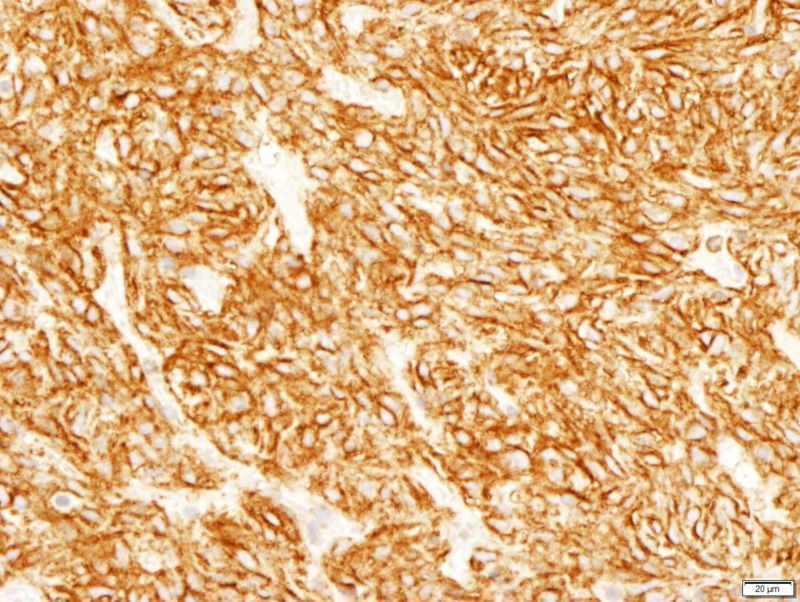
CD 99 positive CD: cluster of differentiation

**Figure 5:**
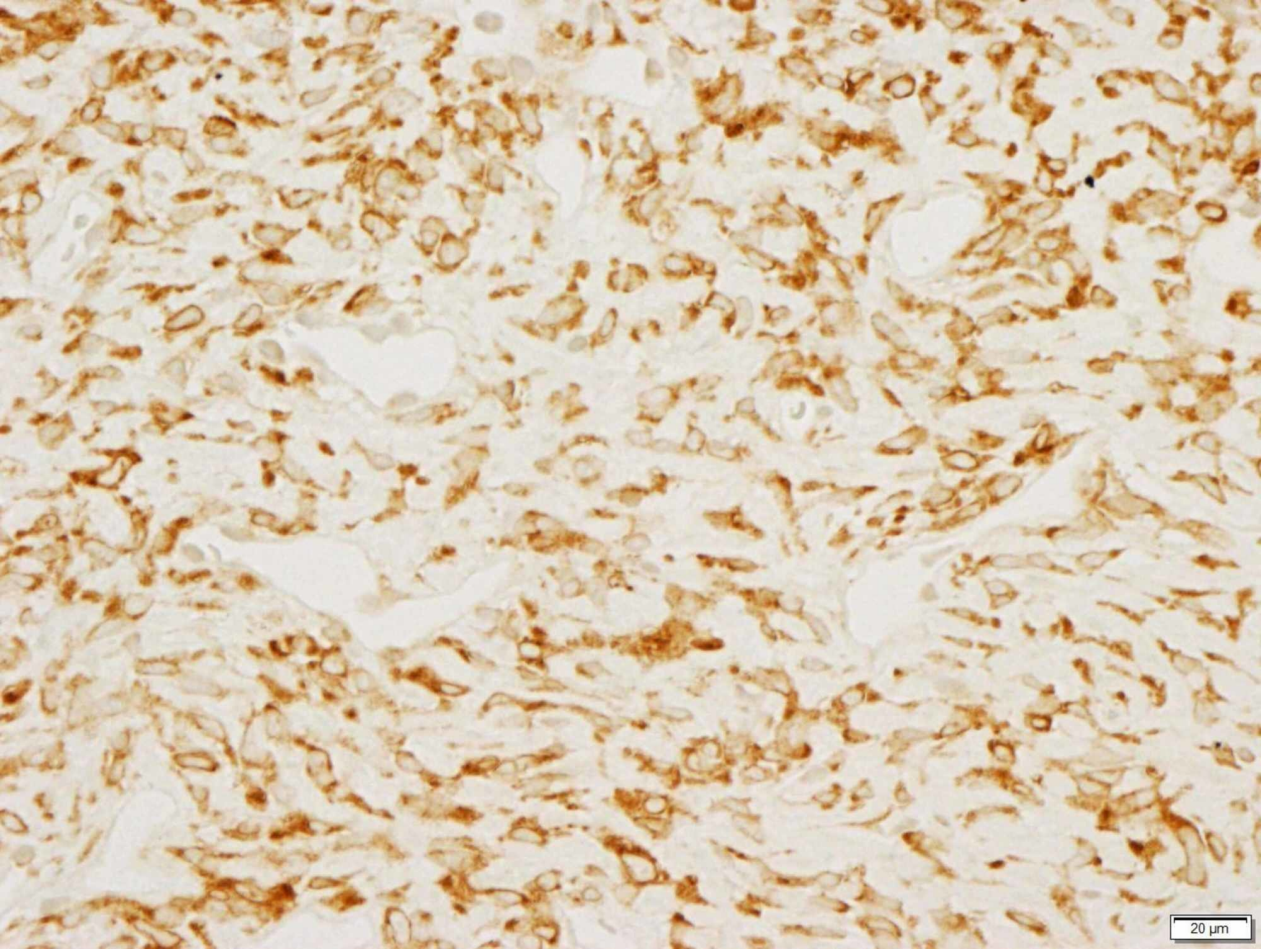
BCL-2 positive BCL-2: B-cell lymphoma

**Figure 6:**
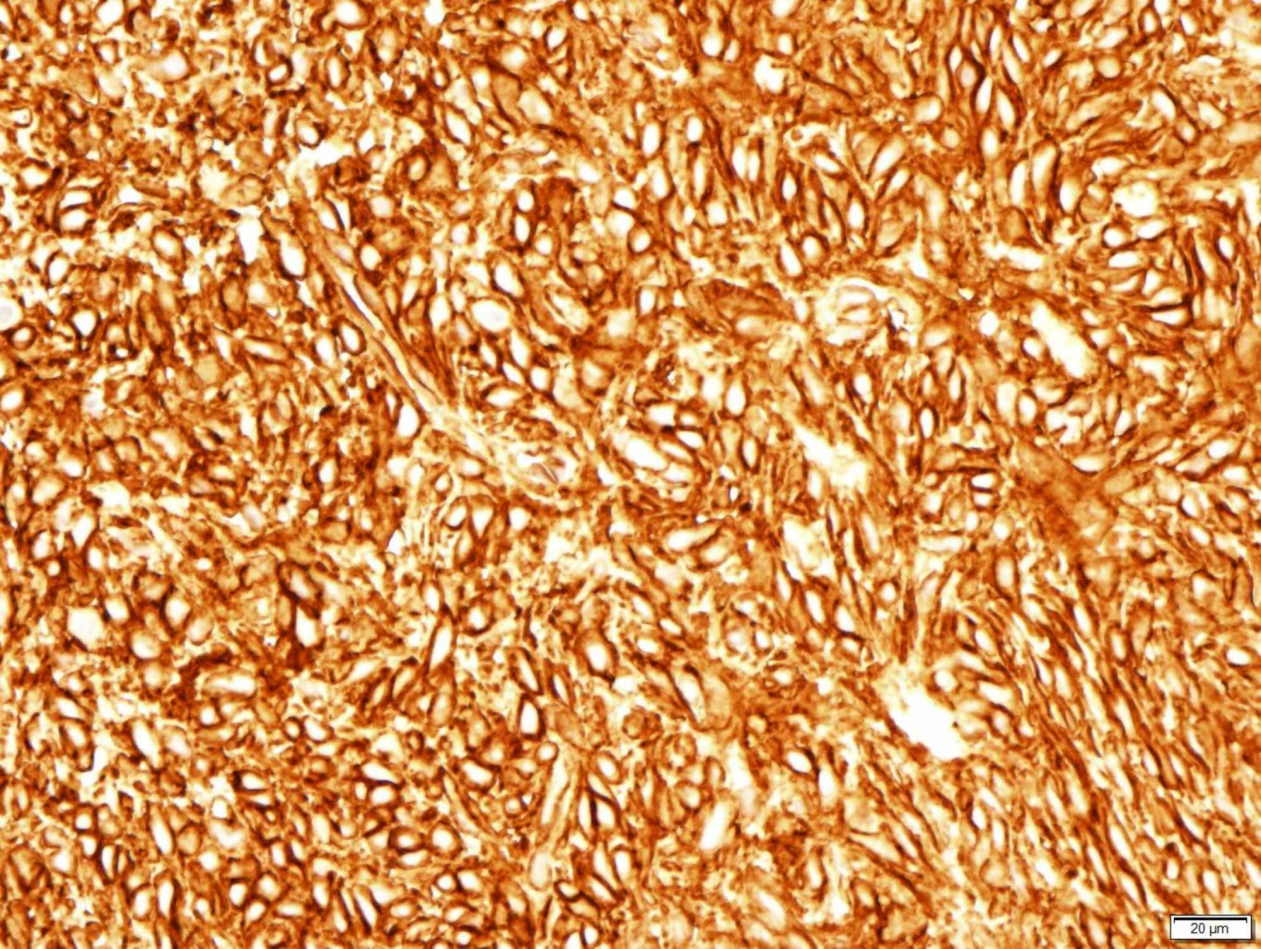
CD 34 positive CD: cluster of differentiation

All patients underwent an orbitotomy and an excisional biopsy. Intraoperatively, there was a well-encapsulated vascularised mass. There were two cases (cases 4 and 5) whereby the tumour was inadvertently ruptured intra-operatively. However, no other complications were encountered. The tumours were excised and sent for histopathological examination. Histologically, findings of spindle cell fascicles that were in a non-specific arrangement and thin-walled blood vessels within the tumour were present in all cases (Figures 2-3). Samples were stained with various immunohistochemistry stains (Figures 4-6) All patients were consistently positive for cluster of differentiation 34 (CD34). Immunohistochemistry results for all seven patients are summarized in Table 2.

**Table 2:** Immunohistochemistry staining and incidence of recurrence CD: cluster of differentiation; BCL-2: B-cell lymphoma; EMA: epithelial membrane antigen; AE: anion exchanger; SMA: smooth muscle actin

CASES	IMMUNOHISTOCHEMISTRY STAINING	MARGINS CLEARED	RECCURRENCE
Case 1	CD34: positive, diffusely and strong intensity	No	Yes
	CD99: positive, diffusely and strong intensity		
	BCL-2, S-100: positive		
	EMA/AE1/AE3/CD3: moderate intensity		
Case 2	CD34: positive	No	Yes
	Vimentin: positive		
	BCL-2, S-100: positive		
Case 3	CD34: strong positive	Yes	No
	CD99: positive		
	EMA: weak positive		
	S100: strong positive		
Case 4	CD34: positive	Yes	No
	CD99: positive		
	SMA: positive in some of the cells.		
Case 5	CD34: positive, diffusely and strong intensity	Yes	No
Case 6	CD34: positive, diffusely and strong intensity	Yes	No
	CD99: positive, diffusely and strong intensity		
	BCL-2: S-100: positive		
Case 7	CD34: positive, diffusely and strong intensity	No	Yes
	CD99: positive		
	S-100: positive, diffusely and strong intensity		
	BCL-2: focal positive		

Surgical margins were not cleared in three of the cases; recurrence of the disease was seen in all three cases within two years postoperatively. All seven patients had regular follow-ups. Patients with tumour margin clearance were followed up yearly while patients without tumour margin clearance were followed up more frequently. Among the cases with recurrences, there was no malignant transformation of the tumour among them.

## Discussion

An orbital solitary fibrous tumour has been reported to predominantly occur in the middle-aged group, although the age of presentation can range from 20-76 years [3]. The patients in our study comprise a younger age group. Studies have shown the condition to have no gender preference [4-6]. Patients commonly present with unilateral, slowly progressive, painless proptosis with a well-defined, enhancing mass on computed tomography (CT)/magnetic resonance imaging (MRI) scan - this was demonstrated by our patient sample. Periodically, patients can be found to have visual disturbances, ocular motility restriction or blepharoptosis [6]. Neighbouring bone and intracranial extension are rare [3].

Several other intraorbital neoplasms, such as fibrous histiocytoma, nerve sheath meningioma, haemangiopericytoma and schwannoma, share a similar course and imaging reports orbital SFT. This makes it difficult to distinguish these tumours clinically.

The classic histopathological feature of SFT is the presence of spindle cells growing in a haphazard manner in the cellular stroma, commonly known as the “patternless pattern” [5-6]. Another feature published in the literature, demonstrated by our patients, was the numerous branching of thin-walled vessels of different sizes - the staghorn pattern [7-8]. Other features include thick bands of collagen interspersed between tumour cells and alternating hypo- and hypercellular areas [3,7,9].

Immunohistochemical studies specific to SFT include strong and diffuse positivity to CD34, vimentin and BCL-2 and non-specific reactivity to CD99 [7,9-10]. SFTs are negative to desmin, cytokeratin, factor VIII-related antigen, S-100, smooth muscle actin (SMA), and muscle-specific actin [3,9-10]. Such factors are important in distinguishing the usually benign nature of orbital SFT from other mesenchymal orbital tumours, especially haemangiopericytoma, due to its malignant nature and aggressive behaviour [9]. CD34 was diffuse and strongly positive in all our patients. Five cases stained positive for CD99 and four others for BCL-2.

Though five patients had positive staining for S-100, the strong positive staining of CD34 and the presence of other positive staining were supportive of the diagnosis of SFT. Interestingly, a reported case series with patients that showed negative staining for S-100 had patients from an older age group [3,11-12]. However, a case report of a 22-year-old patient showed equivocal results for S-100 staining [6]. The patients in our study were generally younger, whereby five out of seven patients were S-100 positive.

Most orbital SFTs behave in a benign manner, although one case of malignant orbital SFT has been reported [13]. Histologically, the features of nuclear atypia, increased cellularity, necrosis and greater than 4 mitoses/10 high-power fields are suggestive of malignant transformation [4,9]. Mainstay treatment is complete excision of the tumour. Local invasion and recurrence are associated with incomplete excision of the tumour [3,7,14].

Among three patients whose tumours did not have clear margins, all developed recurrence. It is not always possible to achieve a bloc surgical excision with complete tumour removal. Therefore, patients with residual tumours require regular follow-up and extensive counselling regarding the possibility of a second surgery. Among the patients who had a recurrence, all required a second surgery to remove the recurrent tumours.

## Conclusions

In conclusion, orbital solitary fibrous tumours are believed to have been underdiagnosed in the past. Recently, however, with the advancement in immunohistochemical staining, SFT distinction with other more aggressive tumours have been identified, making the diagnosis of the tumour more conclusive. This is necessary to ensure that correct diagnosis and appropriate management are achieved.
